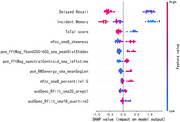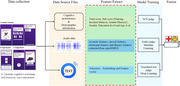# A Multimodal Fusion Framework for Early Non‐Invasive Screening of Cognitive Impairment Using Language Digital Biomarkers

**DOI:** 10.1002/alz70856_099209

**Published:** 2025-12-24

**Authors:** Jiahui Xu, Zhixing Zhou, Huanhuan Xia, Nan Chen, Quan Chen

**Affiliations:** ^1^ University of Shanghai for Science and Technology, Shanghai, China; ^2^ Shanghai Bestcovered Limited, Shanghai, China

## Abstract

**Background:**

Alzheimer's disease (AD) is a prevalent neurodegenerative condition, and its early diagnosis is critical for timely intervention and treatment. Current diagnostic methods, such as biomarker detection and neuroimaging, are costly and reliant on specialized resources, limiting their accessibility. Non‐invasive cognitive screening, while promising, is often influenced by subjective and environmental factors, reducing its accuracy in practical use. Language biomarker analysis has emerged as a stable and convenient alternative. Advancements in machine learning, particularly the Bidirectional Encoder Representations from Transformers (BERT) model, provide robust support for speech‐based AD screening and hold promise for breakthroughs in early diagnosis.

**Method:**

This study adopted a systematic and scientific method to accurately distinguish between people with cognitive impairment and healthy controls (HC). In terms of data processing, with the approval of the hospital ethics committee, 300 subjects were selected from the C ‐ PAS cohort and the data sets were reasonably divided. Semantic features were obtained through Shanghai cognitive screening (SCS) test, and audio features were extracted using BERT and OpenSMILE. During model training, an SCS score of 84.75 was determined as the classification boundary. For text, a CNN model was constructed, and for audio, five models such as RF and XGBoost were trained. The hard voting method was used for result fusion, and professional indicators such as Specificity were used for evaluation to ensure the reliability and validity of the study.

**Result:**

The proposed framework achieved an accuracy of 91.80%, surpassing the 77.17% accuracy of the MoCA‐Basic test for identifying people with cognitive impairment. Additionally, it attained an F1‐score of 91.85%. Feature importance analysis revealed key biomarkers linked to cognitive impairment, including increased pause time and spectral changes in acoustic features, along with reduced semantic complexity in translated text.

**Conclusion:**

The proposed multimodal framework offers a highly accurate, cost‐effective, and non‐invasive method for the early detection of cognitive impairment. The identified biomarkers provide valuable insights into early functional deficits associated with cognitive decline, advancing our understanding of the disease and enabling the development of more effective screening tools.